# The Phenolic Contents and Antioxidant Activities of Infusions of *Sambucus nigra* L.

**DOI:** 10.1007/s11130-016-0594-x

**Published:** 2017-01-13

**Authors:** Agnieszka Viapiana, Marek Wesolowski

**Affiliations:** Department of Analytical Chemistry, Medical University of Gdansk, Gen. J. Hallera 107, 80-416 Gdansk, Poland

**Keywords:** Elder teas, Phenolic acids, Flavonols, Antioxidant activity

## Abstract

The aim of this work was to evaluate the antioxidant potential of teas prepared from twenty-four commercially available berries and flowers of *Sambucus nigra* L. in relation to their phenolic profile, as reflected by the most representative phenolic acids (caffeic, chlorogenic, *p*-coumaric, ferulic, gallic and syringic acids); flavonols (quercetin, kaempferol, myricetin and rutin); and total phenolic (TPC), phenolic acid (TAC) and flavonoid (TFC) contents. The infusions prepared from elderflowers contained more abundant phenolic compounds than the elderberry infusions. The TPC of these infusions ranged from 19.81 to 23.90 mg of gallic acid equivalents/g dry weight of sample (GAE/g DW) for elderberries and from 15.23 to 35.57 mg GAE/g DW for elderflowers, whereas the TFC ranged from 2.60 to 4.49 mg of rutin equivalents/g dry weight of sample (RUTE/g DW) in elderberry infusions and from 5.27 to 13.19 mg RUTE/g DW in elderflower infusions. Among the phenolic compounds quantified in this study, quercetin (2.07–9.48 mg/g DW) and myricetin (1.17–9.62 mg/g DW) had the highest concentrations in the teas prepared from berries and flowers, respectively. Moreover, the antioxidant potential of elder infusions assessed by 2,2-diphenyl-1-picrylhydrazyl (DPPH) radical and ferric reducing antioxidant power (FRAP) assays revealed that the teas prepared from flowers had higher mean DPPH and FRAP activities than the teas prepared from berries. Therefore, elder beverages could be important dietary sources of natural antioxidants that contribute to the prevention of diseases caused by oxidative stress.

**Electronic supplementary material:**

The online version of this article (doi:10.1007/s11130-016-0594-x) contains supplementary material, which is available to authorized users.

## Introduction

*Sambucus nigra* L., also called elder or European elder, is a widespread species of the *Caprifoliaceae* family that grows in most parts of Europe, West Asia, North Africa, and the USA [1]. Elder cultivars are used as beverages and food flavoring, and the berries have been utilized as dietary supplements in many parts of the world [2]. Nearly every part of the elder plant has some culinary use [3]. Elder fruits or juices may be used in the processing of jams or jellies, and several wineries produce wine from the fruit. Elderberries can also be used in the preparation of pies, punches and liqueurs; the flowers are often added to the batter used to make foodstuffs such as muffins, pancakes or waffles; and the flowers and panicles are used to flavor wines and can also be employed to make tea or non-alcoholic cordial [3]. When marinated, the flower buds are sometimes used as a substitute for capers. In contrast to the berries and flowers, elder leaves and stems are considered toxic and must be avoided in human consumption, whereas the young shoots, when cooked, have a taste similar to asparagus. The older, green parts, however, are also toxic [3]. The elderberries and elderflowers are also useful in folk medicine [4], and the consumption of the juice may contribute to the prevention of several degenerative diseases such as cancer, inflammatory and cardiovascular diseases, and diabetes [1].

The applications of *Sambucus nigra* can be attributed to its characteristic chemical composition, which includes essential oils, free fatty acids, flavonoids and their glycosides, phenolic acids, carotenoids, vitamins and minerals [2]. Phenolic acids and flavonols, along with anthocyanins, constitute the principal secondary metabolites of elder. These non-anthocyanin phenolic constituents have potent antioxidant activities both *in vitro* and *in vivo* due to their reducing properties [5, 6]. Phenolic compounds exhibit various biological activities, such as anti-inflammatory, antiviral, antiallergic, vasoprotective and anti-carcinogenic activities [7]. Recent studies have also revealed the beneficial properties of chlorogenic acid (CGA), including antioxidative, hepatoprotective and hypoglycemic activities; the promising activity of gallic acid (GA) in the treatment of Alzheimer’s disease due to its antioxidant activity; and many biological activities of rosmarinic acid, such as HIV-1 inhibition and antitumor and antihepatitis activities. Therefore, the objective of this study was to evaluate the antioxidant potential (2,2-diphenyl-1-picrylhydrazyl (DPPH) and ferric reducing antioxidant power (FRAP) assays) of teas prepared from commercial elder berries and flowers in relation to their phenolic profile, as reflected by the most representative phenolic acids (caffeic (CA), CGA, *p*-coumaric (*p*CA), ferulic (FA), GA and syringic (SA)) and flavonols (quercetin (Q), kaempferol (K), myricetin (M) and rutin (RUT)) and the total phenolic (TPC), total phenolic acid (TAC) and total flavonoid (TFC) contents. This study was expected to reveal the extent to which elder teas are potential sources of natural antioxidants in comparison to other commonly consumed teas and fruits.

## Materials and Methods

### Plant Material

Twenty-four samples of commercial elder products consisting of 11 berries and 13 flowers were used in this study. These samples were obtained from the herbal enterprises in Poland and Serbia specified in Table 1 and were used for analysis as received, without additional pulverization or drying.

**Table 1:** Characteristics of the analyzed berries and flowers

No.	Herbal product	Herbal enterprise	Confection
Elder berries (whole dried berries)			
1	Elderberry fruit, Ecological tea	Dary Natury/Grodzisk^a^	loose
2	Elderberry fruit, *Sambuci fructus*	Flos/Mokrsko^a^	loose
3	Elderberry fruit, *Sambuci fructus*	Flos/Mokrsko^a^	loose
4	Elderberry fruit, *Sambuci fructus*	Flos/Mokrsko^a^	loose
5	Elderberry fruit, Fruit tea	Herbapol/Kraków^a^	loose
6	Elderberry fruit, Dietary supplement	Kawon/Gostyń^a^	loose
7	Elderberry fruit, Dietary supplement	Kawon/Gostyń^a^	loose
8	Elderberry fruit, Dietary supplement	Kawon/Gostyń^a^	loose
9	Elderberry fruit, Dietary supplement	Kawon/Gostyń^a^	loose
10	Elderberry fruit, Dietary supplement	Kawon/Gostyń^a^	loose
11	Elderberry fruit, Dietary supplement	Kawon/Gostyń^a^	loose
Elder flowers (crumbled dried flowers)			
12	*Sambuci* flos, Zova čaj	Institut za proučavanje bija “Dr. Josif Pančić”^b^	bags
13	*Sambuci* flos, čaj od cvetova zove	Institut za proučavanje bija “Dr. Josif Pančić”^b^	loose
14	*Sambuci* flos, čaj od cvetova zove	Adonis^b^	bags
15	*Sambuci* flos, čaj od cvetova zove	Adonis^b^	loose
16	*Sambuci* flos	Adonis^b^	loose
17	*Sambuci* flos	Beligor/Svrljig^b^	loose
18	Zova, biljni čaj od cvetova zove	Macval/Novi Sad^b^	bags
19	Zova, biljni čaj od cvetova zove	Welton/Osecina^b^	bags
20	Elderberry flower, ecological tea	Flos/Mokrsko^a^	loose
21	Elderberry flower, ecological tea	Dary Natury/Grodzisk^a^	loose
22	Elderberry flower, fix tea	Herbapol/Kraków^a^	bags
23	Elderberry flower, *Sambuci* flos	Kawon/Gostyń^a^	bags
24	Elderberry flower, *Sambuci* flos	Kawon/Gostyń^a^	bags

Country of origin: ^a^ Poland, ^b^ Serbia

### Reagents

Analytical-grade methanol, ethanol, aluminum chloride hexahydrate, ferrous sulfate, sodium molybdate, sodium nitrite, sodium hydroxide, sodium carbonate, hydrochloric acid (0.5 mol/L), Folin-Ciocalteu reagent, DPPH, 6-hydroxy-2,5,7,8-tetramethylchroman-2-carboxylic acid (Trolox) and 2,4,6-tris-(2-pyridyl)-1,3,5-triazine (TPTZ) were obtained from POCh (Gliwice, Poland). HPLC-grade acetonitrile and trifluoroacetic acid (TFA) were supplied by Avantor (Central Valley, PA, USA) and Sigma Aldrich (St. Louis, MO, USA), respectively. Standards of phenolic acids and flavonols were acquired from ChromaDex (Santa Ana, CA, USA). Redistilled water was obtained through the triple distillation of water in a Destamat® Bi-18 system (Heraeus Quarzglas, Hanau, Germany).

### Sample Preparation

The infusions obtained from the berries and flowers were analyzed for their TPC, TAC, TFC and individual phenolic acids (CA, CGA, *p*CA, FA, GA and SA) and flavonols (K, M, Q and RUT). Tea infusions were prepared according to the recipe on the packaging. Overall, 1 g of accurately weighed plant material was infused with 200 mL of boiling water. The infusions were left at room temperature for 15 min and then filtered through Whatman No. 541 filter paper (Macherey-Nagel, Düren, Germany). Before chromatographic analysis, the infusions were filtered again through a 0.45 μm nylon membrane filter (Witko, Łódź, Poland).

### Determination of Total Phenolic, Phenolic Acid and Flavonoid Contents

The TPC was quantified using the Folin-Ciocalteu method [8]. The absorbance at 760 nm was measured on a Metertech UV/Vis spectrophotometer SP-870 (Seoul, South Korea), and the TPC was expressed as milligrams of gallic acid equivalents per gram dry weight of sample (mg GAE/g DW). The TAC was determined at 490 nm according to the Arnov method [9], and the results were expressed as milligrams of caffeic acid equivalents per gram dry weight of sample (mg CAE/g DW). The aluminum chloride method was used for the TFC determination at 510 nm [10], and the results were expressed as milligrams of rutin equivalents per gram dry weight of sample (mg RUTE/g DW).

### Determination of Phenolic Compounds

Phenolic acids and flavonols were separated and quantified using an HPLC-UV system (LaChrom, Merck, Darmstadt, Germany) consisting of an L-7100 pump, an L-7360 column compartment and a μL-7420 UV detector [11]. The separation was carried out at 35 °C on a Hypersil Gold C18 column (250 mm × 4.6 mm, i.d. 5 μm). The mobile phase consisted of solvent A (0.2% TFA in methanol) and solvent B (0.2% TFA in water). The optimized gradient elution consisted of the following program: 5–5% A (0–5 min), 5–25% A (5–40 min), 25–35% A (40–50 min), 35–35% A (50–55 min), 35–65% A (55–60 min) and 65–65% A (60–65 min). The injection volume was 20 μL, and the absorbance was monitored at 280 nm (GA and SA), 320 nm (CA, CGA, *p*CA and FA), and 370 nm (K, M, Q and RUT). The analytes were identified based on comparison of their chromatographic retention times with those of standard compounds. For quantitation, 10 analytical curves were prepared using standard solutions that covered a concentration range of 10 to 100 μg/mL. The concentrations of phenolic compounds in the elder infusions were calculated as mg/g DW.

### Determination of Antioxidant Activity

The method proposed by Tuberoso et al. [12] was used to measure the radical scavenging activity of elder teas using DPPH reagent. The results were calculated as millimoles of Trolox equivalents per gram dry weight of sample (mmol TE/g DW). The FRAP of the teas was estimated according to the method proposed by Benzie and Strain [13], and the results were calculated as millimoles of Fe^2+^ per gram dry weight of sample (mmol Fe^2+^/g DW).

### Statistical Analysis

Three infusions of each sample were prepared, and the quantitation of phenolic compounds and estimation of the antioxidant potential were carried out in triplicate. The results, expressed as the mean values and standard deviations (SD), were analyzed by ANOVA with Duncan’s *post hoc* procedure, Student’s *t*-test and Pearson’s correlation analysis using Statistica software, ver. 10 (StatSoft, Krakow, Poland), and *p* < 0.05 was considered to be statistically significant.

## Results and Discussion

### Phenolic Compounds

ANOVA was used to determine statistically significant differences in the consistency (loose or in bags) and origin (from Poland or Serbia) of the elderflower samples (Table 1) based on the individual phenolic compound concentrations, TPC, TAC, TFC and antioxidant potency (DPPH and FRAP assays) of the elderflower infusions. The infusions prepared from the loose and bagged samples differed only in the levels of CA (*p* = 0.002143) and *p*CA (*p* = 0.012276), whereas the infusions obtained from Polish and Serbian samples differed only in the level of M (*p* = 0.021365).

Quantitation of the TPC, TAC and TFC in the twenty-four commercial elder samples and ANOVA revealed that the mean TPC, TFC and TAC in the teas prepared using flowers were statistically higher than those prepared using berries. The TPC of these infusions ranged from 19.81 to 23.90 mg GAE/g DW for the elderberries and from 15.23 to 35.57 mg GAE/g DW for the flowers. Generally, the infusions from elderflowers contained a slightly higher TPC (27.02 mg GAE/g DW) than the infusions prepared from elderberries (21.81 mg GAE/g DW). The TPCs obtained in this study are comparable to those determined in Jordanian plants (~20 mg GAE/g DW) [14]. Duymuş et al. [15] found higher TPCs in elderberry infusions (67.15 mg GA/g DW), whereas Katalinic et al. [16] and Veljković et al. [17] detected TPCs in flower infusions of 498 mg CAE/L and 42.67 g GAE/kg DW, respectively. Lee and Finn [2] and Akbulut et al. [18] determined TPCs in methanolic extracts of elderberries of 327–582 mg GAE/g berries and 371–432 mg GAE/100 g fresh weight (FW), respectively. A literature search also revealed that the efficiency of phenolic extraction depends on the extraction method, solvent type [19], and drying process used for the plant material [20]. The temperature of the water used in the tea preparation is also crucial. The extraction yield when using hot water differs significantly from that obtained when using water at other temperatures [21].

Flavonoids are secondary metabolites present in plants, and studies on the variation in their contents are important because the biological effects of many plant materials depend on these compounds [22]. The TFC ranged between 2.60 and 4.49 mg RUTE/g DW in the elderberry infusions and from 5.27 to 13.19 mg RUTE/g DW in the elderflower infusions. The largest difference was observed between the mean TFC in the berries and flowers of 3.81 mg RUTE/g and 10.32 mg RUTE/g, respectively. A similar TFC in berries (38.26 mg RUTE/100 g FW) was reported by Anton et al. [23], whereas the TFC in elderflower infusions was determined to be 29.07 g catechin/kg DW [17].

Phenolic acids are a group of secondary metabolites that are widely distributed in plants, and several studies have reported their inhibitory effect on the growth of pathogens and cancer cells [22]. The TAC varied from 1.31 to 3.22 mg CAE/g DW in the elderberry infusions and from 1.19 to 6.52 mg CAE/g DW in the elderflower infusions. To the best of our knowledge, this is the first report of the TAC in elder infusions. As a comparison, Martins et al. [24] obtained 32.69 mg total phenolic acids/g in infusions prepared from thyme and 22.89 mg/g in oregano infusions [25].

Regarding the levels of individual phenolic compounds, flavonols were generally detected in elder teas at higher concentrations than phenolic acids. ANOVA showed that the elderberry and elderflower infusions differed statistically in their mean contents of *p*CA, RUT, M, Q and K but not in the contents of CA, SA and FA. The berry infusions were significantly richer in Q (2.07–9.48 mg/g DW), RUT (0.44–6.45 mg/g DW), K (0.62–4.98 mg/g DW) and GA (0.32–0.53 mg/g DW) than the flower infusions, which had statistically higher levels of M (1.17–9.62 mg/g DW), CGA (0.31–6.26 mg/g DW) and *p*CA (0.21–0.45 mg/g DW). The sequence of the levels of phenolic compounds was Q > RUT > K > M > SA > CA > GA > FA > *p*CA > CGA in the berry infusions and M > Q > RUT > K > CGA > SA > CA > GA > FA > *p*CA in the flower infusions.

These data show that the levels of RUT and GA in elderflower teas are comparable with those reported in the literature [17]. Dawidowicz et al. [5] determined RUT in ethanol-water (80:20, *v/v*) extracts of berries, flowers and leaves of elder at concentrations of 0.11 g/100 g, 1.33 g/100 g and 0.07 g/100 g, respectively, and Anton et al. [23] determined K in *Sambucus nigra* berries at a low content of 0.23 mg/100 g FW. Q, the most frequently quantified flavonol in plants, was detected in elderberries at 2.37 mg/100 g [23], 331 mg/kg [26], and 29–60 mg/100 g [27] on a fresh weight basis, as well as in elderflower teas at 0.254 g/kg [17]. Of the phenolic acids, CA was quantified in elderflower teas at 0.132 g/kg DW [17], and *p*CA was detected at 0.55 mg/100 g in fresh elderberries [23]. These values are lower than those obtained in the infusions in the present work.

### Antioxidant Activity

The antioxidant potential is a crucial parameter for establishing the health benefits of food products. Various phytochemical components, such as phenolic compounds, have been identified to be responsible for antioxidant properties [23]. Several tests are used to estimate this potential, *e.g*., DPPH and FRAP assays, which are complementary to the same degree. Both tests are recommended as simple, rapid, reproducible and inexpensive tools for measuring the antioxidant activity of plant extracts. Similar to the chemical composition, the antioxidant potential of plants also depends on many extrinsic factors [28], such as storage, soil type, agronomic practices, climatic factors and technological treatments, as well as differences in the cultivars or varying stress conditions of the vegetation [29].

The antioxidant potential of the elder infusions estimated using the DPPH and FRAP assays revealed that the teas prepared from flowers had higher mean DPPH and FRAP activities than those prepared from berries (ANOVA). The DPPH results obtained in this study are similar to those obtained by Buřičivá et al. [29] for elderflower teas (58.8 mg ascorbic acid/g). Veljković et al. [17] also assessed the antioxidant activity of elderflower teas and determined levels of 0.118 mol TE/kg (DPPH test) and 0.402 mol Fe^2+^/kg (FRAP test). For fresh elderberries, lower values than those obtained in this study were found by Akbulut et al. [18] (5.04–6.37 mmol TE/100 g, FRAP assay) and Özgen et al. [30] (10.8 μmol TE/g, DPPH, and 24.1 μmol TE/g, FRAP assay).

Notably, the free-radical scavenging activity of elderflowers was higher than those of other fruits that are well-known for their antioxidant potency [27]. In this study, the DPPH activities of the elderflowers were higher than the published activities of teas prepared from strawberries (0.142 mol/kg), raspberries (0.180 mol/kg) or blueberries (0.140 mol/kg), whereas the FRAP activities were similar to the published activities of the teas prepared from these fruits – 0.417, 1.121 and 0.636 mol Fe^2+^/kg, respectively [17].

### Correlation Analysis

The Pearson’s correlation coefficients listed in Table 2 imply that the TPCs and TFCs strongly correlate with the data obtained from the DPPH and FRAP assays for the elderflower teas but significant correlations were only found between the TPCs and the results of both assays for the elderberry teas. These high correlations are consistent with the view that these two assays share a similar mechanistic basis, viz. the transfer of electrons from the antioxidant to reduce an oxidant [31]. On the other hand, no statistically significant correlations were found between the antioxidant activity and the TAC or individual flavonol and phenolic acid concentrations. In these cases, the correlation coefficients were generally low, *i.e*., below 0.55.

**Table 2:** Pearson’s correlation coefficients between the phenolic compound contents and antioxidant activities of the elder tea infusions

Total contents of phenolic compounds	Antioxidant potential			
	Elderberries		Elderflowers	
	DPPH	FRAP	DPPH	FRAP
TPC	**0.66**	**0.70**	**0.78**	**0.65**
TFC	0.45	-0.10	**0.63**	**0.62**
TAC	0.16	0.18	0.10	0.05

Statistically significant correlations (*p* < 0.05) are in bold

The significant relationships between the antioxidant properties and TPCs in the berry-derived teas and the TPC and TFC in the flower-derived teas imply that the TPC is one of the main factors responsible for the antioxidant potential of elder beverages. This implication was confirmed by Gonçalves et al. [32]*,* who found a high relationship between the antioxidant activity (DPPH assay) and TPC of infusions prepared from Mediterranean medicinal plants. However, the correlations between antioxidant activities and TPCs have been higher in some studies than those obtained in this work. For instance, Rodriguez Vaquero et al. [33] found high correlations between both the FRAP (*r* = 0.81) and DPPH (*r* = 0.86) activities and TPCs of infusions prepared from Argentinian herbs that are commercially available in pharmacies. Jakobek et al. [34] determined the flavonols, phenolic acids and antioxidant activities of red fruits (including elderberries) harvested in Croatia and found a high correlation between the total polyphenol contents and DPPH activities (*r* = 0.98).

Values below 0.55 of the Pearson’s correlation coefficients calculated between the antioxidant activities and individual phenolic compound contents suggest that the constituents that occur separately in the elder teas could not be responsible for the antioxidant properties. However, the DPPH activities strongly correlated with the contents of Q (0.74), CA (0.91) and *p*CA (0.74) in red fruits [34].

## Conclusion

The infusions prepared from elderflowers contained more abundant phenolic compounds than the elderberry infusions. Among the quantified plant metabolites, Q and M were detected in the highest concentrations in the teas prepared from elderberries and elderflowers, respectively. Moreover, the antioxidant potential of the elder infusions assessed by DPPH and FRAP assays revealed that the teas prepared from flowers had higher mean DPPH and FRAP activities than the teas prepared from berries, which was confirmed by the significant relationships obtained between the antioxidant properties and the TPCs and TFCs. The results of this study suggest that elder beverages could be an important dietary source of natural antioxidants for the prevention of diseases caused by oxidative stress.

## Electronic supplementary material

ESM 1 (DOC 70 kb)

ESM 2 (DOC 67 kb)

ESM 3 (DOC 651 kb)

## Abbreviations

DPPH: 2,2-diphenyl-1-picrylhydrazyl
FRAP: Ferric reducing antioxidant power
TAC: Total phenolic acid content
TFC: Total flavonoid content
TPC: Total phenolic content
TPTZ: 2,4,6-tris-(2-pyridyl)-1,3,5-triazine
