# Supplementary material for: The Phenolic Contents and Antioxidant Activities of Infusions of Sambucus nigra L
Source: Plant Foods Hum Nutr. 2017 Jan 13;72(1):82–7. doi: 10.1007/s11130-016-0594-x (PMC5325840; doi:10.1007/s11130-016-0594-x)
Supplement: Supplementary file 1 — (DOC 70 kb) [file 11130_2016_594_MOESM1_ESM.doc]

The phenolic contents and antioxidant activities of infusions of *Sambucus nigra* L. Plant Foods for Human Nutrition. Agnieszka Viapiana and Marek Wesolowski, Department of Analytical Chemistry, Medical University of Gdansk, Gen. J. Hallera 107, 80-416 Gdansk, Poland, *E-mail address*: [marwes@gumed.edu.pl](mailto:marwes@gumed.edu.pl)

**Table 1** Total phenolic (TPC), total phenolic acid (TAC) and total flavonoid (TFC) contents and antioxidant activities (DPPH and FRAP assays) of elder infusions

|  | TPC (mg GAE/g DW) | TAC (mg CAE/g DW) | TFC (mg RUTE/g DW) | DPPH (mmol TE/g DW) | FRAP (mmol Fe^2+^/g DW) |
| --- | --- | --- | --- | --- | --- |
| Elder berries | | | | | |
| 1 | 23.22 ± 2.52^a^ | 3.22 ± 0.83^d^ | 3.45 ± 0.77^ab^ | 0.43 ± 0.06^ac^ | 0.66 ± 0.09^abc^ |
| 2 | 23.23 ± 2.15^a^ | 2.65 ± 0.62^bcd^ | 3.26 ± 0.58^ab^ | 0.33 ± 0.02^bd^ | 0.64 ± 0.09^abc^ |
| 3 | 20.66 ± 3.55^a^ | 2.05 ± 0.08^abc^ | 3.38 ± 0.71^ab^ | 0.37 ± 0.03^ab^ | 0.56 ± 0.06^ab^ |
| 4 | 21.85 ± 1.91^a^ | 2.07 ± 0.25^abc^ | 4.42 ± 0.63^a^ | 0.42 ± 0.02^ac^ | 0.55 ± 0.01^ab^ |
| 5 | 21.33 ± 2.43^a^ | 1.49 ± 0.24^a^ | 4.49 ± 0.46^a^ | 0.43 ±0.01^a^ | 0.57 ± 0.04^ab^ |
| 6 | 22.09 ± 2.02^a^ | 2.01 ± 0.21^abc^ | 4.42 ± 0.49^a^ | 0.52 ± 0.03^ce^ | 0.64 ± 0.05^abc^ |
| 7 | 22.09 ± 2.09^a^ | 1.76 ± 0.14^ab^ | 3.48 ± 0.34^ab^ | 0.55 ± 0.01^e^ | 0.73 ± 0.08^c^ |
| 8 | 19.81 ± 2.30^a^ | 2.36 ± 0.29^abcd^ | 2.60 ± 0.44^b^ | 0.28 ± 0.03^d^ | 0.49 ± 0.01^a^ |
| 9 | 23.90 ± 0.20^a^ | 3.22 ± 0.30^d^ | 3.89 ± 0.42^ab^ | 0.44 ± 0.06^ac^ | 0.66 ± 0.08^bc^ |
| 10 | 20.32 ± 2.71^a^ | 1.31 ± 0.83^a^ | 3.71 ± 0.38^ab^ | 0.37 ± 0.05^ab^ | 0.52 ± 0.07^ab^ |
| 11 | 21.61 ± 2.73^a^ | 3.07 ± 0.17^cd^ | 3.42 ± 0.85^ab^ | 0.39 ± 0.03^ab^ | 0.51 ± 0.08^c^ |
| Elder flowers | | | | | |
| 12 | 22.84 ± 0.61^b^ | 4.11 ± 0.35^cd^ | 8.94 ± 0.36^bcd^ | 0.62 ± 0.06^ab^ | 0.59 ± 0.08^abc^ |
| 13 | 23.43 ± 1.27^bc^ | 4.77 ± 0.14^cde^ | 8.25 ± 0.85^b^ | 0.79 ± 0.02^def^ | 0.79 ± 0.01^bcd^ |
| 14 | 25.05 ± 1.07^bcd^ | 2.19 ± 0.43^b^ | 10.06 ± 0.23^bcde^ | 0.64 ± 0.01^ab^ | 0.50 ± 0.09^ab^ |
| 15 | 26.91 ± 1.53^de^ | 5.12 ± 0.16^de^ | 11.52 ± 0.56^efg^ | 0.74 ± 0.05^cd^ | 0.79 ± 0.09^cd^ |
| 16 | 30.74 ± 1.54^fg^ | 2.68 ± 0.55^b^ | 11.91 ± 0.93^efg^ | 0.73 ± 0.06^cd^ | 1.07 ± 0.01^e^ |
| 17 | 26.81 ± 3.70^de^ | 1.19 ± 0.09^a^ | 10.46 ± 1.20^cdef^ | 0.87 ± 0.06^fg^ | 1.03 ± 0.01^de^ |
| 18 | 32.70 ± 0.71^gh^ | 3.90 ± 0.83^c^ | 11.28 ± 1.92^efg^ | 0.85 ± 0.02^efg^ | 1.04 ± 0.02^de^ |
| 19 | 29.33 ± 0.39^ef^ | 6.52 ± 0.43^f^ | 12.43 ± 0.06^fg^ | 0.75 ± 0.01^cd^ | 1.14 ± 0.01^e^ |
| 20 | 26.39 ± 2.49^cde^ | 4.43 ± 0.99^cde^ | 10.64 ± 0.88^def^ | 0.68 ± 0.06^bc^ | 0.80 ± 0.09^bcd^ |
| 21 | 30.60 ± 1.82^fg^ | 5.23 ± 0.25^e^ | 11.67 ± 1.19^efg^ | 0.79 ± 0.01^def^ | 0.50 ± 0.07^ab^ |
| 22 | 15.23 ± 2.05^a^ | 3.99 ± 0.07^c^ | 5.27 ± 0.66^bc^ | 0.57 ± 0.07^a^ | 0.48 ± 0.01^a^ |
| 23 | 35.57 ± 0.56^h^ | 5.40 ± 0.17^e^ | 13.19 ± 1.79^a^ | 0.92 ± 0.01^g^ | 1.04 ± 0.05^e^ |
| 24 | 25.50 ± 1.95^bcd^ | 4.70 ± 0.41^cde^ | 8.60 ± 0.94^g^ | 0.76 ± 0.01^de^ | 0.73 ± 0.04^abc^ |

The results are expressed as the mean values and standard deviations (SD) (n = 3). The mean values within the same column with different superscripts are significantly different ( p < 0.05).
