# Supplementary material for: The Phenolic Contents and Antioxidant Activities of Infusions of Sambucus nigra L
Source: Plant Foods Hum Nutr. 2017 Jan 13;72(1):82–7. doi: 10.1007/s11130-016-0594-x (PMC5325840; doi:10.1007/s11130-016-0594-x)
Supplement: Supplementary file 2 — (DOC 67 kb) [file 11130_2016_594_MOESM2_ESM.doc]

The phenolic contents and antioxidant activities of infusions of *Sambucus nigra* L. Plant Foods for Human Nutrition. Agnieszka Viapiana and Marek Wesolowski, Department of Analytical Chemistry, Medical University of Gdansk, Gen. J. Hallera 107, 80-416 Gdansk, Poland, *E-mail address*: [marwes@gumed.edu.pl](mailto:marwes@gumed.edu.pl)

**Table 2** Results of quantitation of individual phenolic acids and flavonols (mg/g DW) in elder teas

|  | **GA** | **CGA** | **CA** | **SA** | **pCA** | **FA** | **RUT** | **M** | **Q** | **K** |
| --- | --- | --- | --- | --- | --- | --- | --- | --- | --- | --- |
| Elder berries | | | | | | | | | | |
| 1 | 0.32 ± 0.02^a^ | 0.03 ± 0.01^a^ | 0.25 ± 0.06^a^ | 0.52 ± 0.06^a^ | 0.18 ± 0.02^a^ | 0.29 ± 0.06^a^ | 0.44 ± 0.04^c^ | 1.45 ± 0.23^a^ | 4.34 ± 1.83^a^ | 2.02 ± 0.89^ab^ |
| 2 | 0.50 ± 0.01^ab^ | 0.05 ± 0.01^a^ | 0.65 ± 0.12^bc^ | 0.58 ± 0.05^ab^ | 0.19 ± 0.04^a^ | 0.29 ± 0.04^a^ | 3.79 ± 1.41^ab^ | 0.76 ± 0.14^a^ | 6.04 ± 2.01^a^ | 3.42 ± 1.03^ab^ |
| 3 | 0.45 ± 0.05^ab^ | 0.04 ± 0.01^a^ | 0.62 ± 0.04^b^ | 0.61 ± 0.16^ab^ | 0.20 ± 0.01^a^ | 0.30 ± 0.02^a^ | 2.18 ± 0.54^ac^ | 0.92 ± 0.21^a^ | 5.57 ± 1.67^a^ | 3.24 ± 0.89^ab^ |
| 4 | 0.45 ± 0.02^ab^ | 0.02 ± 0.01^a^ | 0.47 ± 0.15^ab^ | 0.91 ± 0.03^abc^ | 0.19 ± 0.05^a^ | 0.28 ± 0.01^a^ | 6.45 ± 0.91^b^ | 1.22 ± 0.09^a^ | 6.18 ± 1.78^a^ | 3.48 ± 0.78^ab^ |
| 5 | 0.40 ± 0.05^ab^ | 0.03 ± 0.01^a^ | 0.96 ± 0.02^c^ | 0.52 ± 0.02^a^ | 0.17 ± 0.04^a^ | 0.26 ± 0.13^a^ | 3.90 ± 0.53^ab^ | 0.69 ± 0.03^a^ | 2.07 ± 0.67^a^ | 0.65 ± 0.07^a^ |
| 6 | 0.43 ± 0.07^ab^ | 0.04 ± 0.01^a^ | 0.52 ± 0.09^ab^ | 0.59 ± 0.25^ab^ | 0.18 ± 0.07^a^ | 0.28 ± 0.04^a^ | 4.10 ± 1.02^ab^ | 1.06 ± 0.89^a^ | 2.18 ± 0.98^a^ | 1.63 ± 0.09^ab^ |
| 7 | 0.38 ± 0.11^ab^ | 0.03 ± 0.01^a^ | 0.32 ± 0.14^ab^ | 0.78 ± 0.01^abc^ | 0.19 ± 0.03^a^ | 0.28 ± 0.03^a^ | 3.91 ± 0.09^ab^ | 1.18 ± 0.56^a^ | 9.25 ± 1.01^a^ | 0.62 ± 0.07^a^ |
| 8 | 0.48 ± 0.03^ab^ | 0.03 ± 0.02^a^ | 0.21 ± 0.05^a^ | 1.08 ± 0.23^c^ | 0.21 ± 0.02^a^ | 0.32 ± 0.05^a^ | 3.68 ± 1.02^ab^ | 1.42 ± 0.72^a^ | 7.06 ± 0.78^a^ | 3.95 ± 1.05^ab^ |
| 9 | 0.45 ± 0.15^ab^ | 0.04 ± 0.01^a^ | 0.46 ± 0.02^ab^ | 0.55 ± 0.02^a^ | 0.18 ± 0.03^a^ | 0.27 ± 0.02^a^ | 3.43 ± 1.98^a^ | 0.59 ± 0.08^a^ | 9.48 ± 1.76^a^ | 4.98 ± 1.04^b^ |
| 10 | 0.53 ± 0.06^b^ | 0.03 ± 0.01^a^ | 0.21 ± 0.02^a^ | 1.03 ± 0.01^bc^ | 0.21 ± 0.01^a^ | 0.30 ± 0.02^a^ | 4.14 ± 1.32^ab^ | 1.67 ± 0.67^a^ | 4.86 ± 0.62^a^ | 0.68 ± 0.07^a^ |
| 11 | 0.38 ± 0.08^ab^ | 0.04 ± 0.02^a^ | 0.47 ± 0.14^ab^ | 0.55 ± 0.18^a^ | 0.18 ± 0.03^a^ | 0.28 ± 0.01^a^ | 3.33 ± 0.93^a^ | 1.04 ± 0.94^a^ | 4.51 ± 0.99^a^ | 1.47 ± 0.09^ab^ |
| Elder flowers | | | | | | | | | | |
| 12 | 0.35 ± 0.12^abc^ | 0.54 ± 0.11^abc^ | 0.77 ± 0.23^b^ | 0.35 ± 0.08^abcd^ | 0.29 ± 0.01^ab^ | 0.28 ± 0.08^bc^ | 1.23 ± 0.21^bcde^ | 3.28 ± 0.19^b^ | 6.92 ± 1.72^g^ | 0.96 ± 0.21^ab^ |
| 13 | 0.35 ± 0.09^abc^ | 5.68 ± 1.37^f^ | 0.21 ± 0.11^a^ | 0.60 ± 0.12^abc^ | 0.23 ± 0.08^a^ | 0.28 ± 0.06^bc^ | 1.64 ± 0.28^cdef^ | 7.62 ± 0.43^d^ | 4.12 ± 1.83^e^ | 0.79 ± 0.18^ab^ |
| 14 | 0.35 ± 0.10^abc^ | 5.44 ± 0.98^f^ | 0.29 ± 0.06^a^ | 0.59 ± 0.08^abc^ | 0.21 ± 0.09^a^ | 0.30 ± 0.10^bc^ | 1.70 ± 0.12^def^ | 6.86 ± 0.23^d^ | 5.29 ± 1.93^f^ | 1.02 ± 0.09^b^ |
| 15 | 0.36 ± 0.09^abc^ | 0.35 ± 0.10^a^ | 1.05 ± 0.06^c^ | 0.65 ± 0.14^bcde^ | 0.45 ± 0.10^d^ | 0.29 ± 0.09^bc^ | 5.40 ± 0.20^g^ | 1.99 ± 0.11^a^ | 1.13 ± 0.47^ab^ | 0.92 ± 0.21^ab^ |
| 16 | 0.32 ± 0.03^ab^ | 0.61 ± 0.21^abc^ | 1.50 ± 0.09^d^ | 0.71 ± 0.21^cde^ | 0.23 ± 0.06^a^ | 0.49 ± 0.12^e^ | 0.57 ± 0.13^ab^ | 6.92 ± 0.32^d^ | 1.06 ± 0.11^ab^ | 0.85 ± 0.11^ab^ |
| 17 | 0.31 ± 0.09^a^ | 0.84 ± 0.08^cd^ | 0.31 ± 0.02^a^ | 0.57 ± 0.08^ab^ | 0.25 ± 0.09^ab^ | 0.25 ± 0.08^ab^ | 2.25 ± 0.17^f^ | 1.44 ± 0.24^a^ | 2.60 ± 0.43^cd^ | 0.78 ± 0.23^ab^ |
| 18 | 0.38 ± 0.12^abc^ | 1.05 ± 0.97^d^ | 0.37 ± 0.07^a^ | 0.65 ± 0.32^bcde^ | 0.23 ± 0.08^a^ | 0.30 ± 0.13^bc^ | 1.80 ± 0.10^ef^ | 1.37 ± 0.15^a^ | 0.55 ± 0.07^a^ | 1.01 ± 0.43^b^ |
| 19 | 0.39 ± 1.05^bc^ | 0.83 ± 0.56^bcd^ | 0.21 ± 0.01^a^ | 0.77 ± 0.13^e^ | 0.27 ± 0.10^ab^ | 0.31 ± 0.09^cd^ | 0.78 ± 0.21^abc^ | 1.50 ± 0.93^a^ | 0.67 ± 0.11^a^ | 0.71 ± 0.09^ab^ |
| 20 | 0.34 ± 0.15^abc^ | 0.35 ± 0.07^a^ | 1.35 ± 0.78^d^ | 0.67 ± 0.17^bcde^ | 0.41± 0.12^cd^ | 0.28 ± 0.06^bc^ | 1.09 ± 0.24^abcd^ | 9.62 ± 1.48^f^ | 0.51 ± 0.03^a^ | 0.91 ± 0.07^ab^ |
| 21 | 0.40 ± 0.20^c^ | 2.45 ± 0.68^e^ | 0.75 ± 0.11^b^ | 0.74 ± 0.42^de^ | 0.33 ± 0.09^bc^ | 0.35 ± 0.21^d^ | 2.08 ± 0.90^ef^ | 5.66 ±1.92^c^ | 2.93 ± 0.98^d^ | 1.01 ± 0.63^b^ |
| 22 | 0.35 ± 0.21^abc^ | 0.43 ± 0.09^abc^ | 0.34 ± 0.08^a^ | 0.51 ± 0.18^a^ | 0.21 ± 0.06^a^ | 0.22 ± 0.11^a^ | 0.26 ± 0.08^a^ | 1.17 ± 0.09^a^ | 0.89 ± 0.43^ab^ | 0.55 ± 0.03^a^ |
| 23 | 0.32 ± 0.89^ab^ | 0.42 ± 0.07^ab^ | 0.33 ± 0.09^a^ | 0.69 ± 0.15^bcde^ | 0.22 ± 0.07^a^ | 0.25 ± 0.08^ab^ | 1.76 ± 0.28^ef^ | 7.83 ± 1.73^de^ | 0.50 ± 0.08^a^ | 0.57 ± 0.09^a^ |
| 24 | 0.37 ± 0.97^abc^ | 6.26 ± 1.41^g^ | 0.23 ± 0.06^a^ | 0.62 ± 0.12^abcd^ | 0.23 ± 0.09^a^ | 0.31 ± 0.17^cd^ | 1.85 ± 0.32^ef^ | 8.65 ± 1.63^e^ | 1.72 ± 0.78^bc^ | 1.11 ± 0.53^b^ |

The results are expressed as the mean values and standard deviations (SD) (n = 3). The mean values within the same column with different superscripts are significantly different ( p < 0.05).
