# Supplementary material for: The Phenolic Contents and Antioxidant Activities of Infusions of Sambucus nigra L
Source: Plant Foods Hum Nutr. 2017 Jan 13;72(1):82–7. doi: 10.1007/s11130-016-0594-x (PMC5325840; doi:10.1007/s11130-016-0594-x)
Supplement: Supplementary file 3 — (DOC 651 kb) [file 11130_2016_594_MOESM3_ESM.doc]

The phenolic contents and antioxidant activities of infusions of *Sambucus nigra* L. Plant Foods for Human Nutrition. Agnieszka Viapiana and Marek Wesolowski, Department of Analytical Chemistry, Medical University of Gdansk, Gen. J. Hallera 107, 80-416 Gdansk, Poland, *E-mail address*: [marwes@gumed.edu.pl](mailto:marwes@gumed.edu.pl)

**Figure 1** HPLC profile of infusions prepared from elderberries (A) and elderflowers (B) of *Sambucus nigra* L. Phenolic acids: caffeic (CA), chlorogenic (CGA), *p*-coumaric (*p*CA), ferulic (FA), gallic (GA) and syringic (SA) and flavonols: kaempferol (K), myricetin (M), quercetin (Q) and rutin (RUT).
